# The potential of community-based radiographic screening to end TB in India

**DOI:** 10.1371/journal.pgph.0004716

**Published:** 2025-06-24

**Authors:** Sandip Mandal, Manoj Toshniwal, Nimalan Arinaminpathy, Kiran Rade, Puneet Dewan, Raghuram Rao, Urvashi B Singh

**Affiliations:** 1 John Snow India, New Delhi, India; 2 Independent consultant, India; 3 Imperial College London, United Kingdom; 4 The Gates Foundation, Seattle, United States of America; 5 Central TB Division, Government of India, New Delhi, India; PLOS: Public Library of Science, UNITED STATES OF AMERICA

Despite major global initiatives such as End TB strategy, progress in the global TB response has remained slow, and even reversed following the COVID-19 pandemic [1]. Recent, adverse developments in global health financing are likely to pose further greater challenges in coming years. However in India, with the highest absolute TB burden globally, major initiatives were recently developed and broadly deployed. These include massive efforts to expand access to rapid diagnostics, to engage private healthcare providers [2], to use community health workers to screen vulnerable populations for TB symptoms, and to expand use of preventive therapy for household contacts and vulnerable populations [3]. India has also embarked on a major study to explore prevention offered by adult BCG revaccination amongst vulnerable populations. Critical as these initiatives are, more will be needed to address all sources of transmission, in a sustained way. Moreover, while the vaccine pipeline is promising, the prospect of mass immunisation with a highly effective vaccine is uncertain and, in the best case, is still years away. With slow-moving interventions that may take decades to yield results, how then is India (and indeed any high-burden country), going to reach fast-approaching country targets that are key to ending TB?

We argue that mass screening and treatment would be the quickest, most effective, and least expensive approach to achieve rapid reductions in disease burden. Although such measures would require high initial capital expenditure, they carry several justifications in epidemiological, programmatic and implementation terms, as follows:

*There is a large proportion of infectious TB outside the healthcare system*: In India’s recent national TB prevalence survey, 52% of bacteriologically positive TB did not report symptoms (‘asymptomatic’ TB) while a further 17% had symptoms, but had not yet sought care [4], proportions that are typical of prevalence surveys in other high-burden settings [5]. The latter proportion results from a combination of factors, including low prioritisation of health and low risk perception, with symptoms being accepted as routine. While intensive, patient-sensitive risk communication will be important in reaching this population, it must also be coupled with aggressive, proactive measures to detect TB among those who have not yet sought health care.

*Asymptomatic TB may have an important role in driving TB transmission*: The importance of asymptomatic TB has been gaining increasing recognition [6]. Recent studies have highlighted the complex natural history of TB, with, for example, almost a third of people with infectious, asymptomatic TB remaining without symptoms over a five year period [7]. Such individuals very likely contribute substantially to transmission, without ever being visible to case-finding efforts that rely on symptoms [8].

*Mass screening and treatment may rapidly accelerate declines in disease burden.* Mass screening in Kolin, Czechoslovakia, accompanied a 75% reduction in notifications over a 12-year period [9]. In the Russian Federation, a substantial proportion of the population has routinely undergone screening with chest-X-ray each year, with an attendant, sustained decline in TB incidence over the past decade [1]. In Cape Town, South Africa, the decrease in TB notification rates between 1950 and 1970 happened while 10% of the population were screened annually by mass miniature radiography [10]. More recently, in Vietnam, mass sputum testing with Xpert, and immediate initiation of all positive cases on TB treatment, was associated with prevalence being reduced by almost a half over four years [11].

*Mass screening is more feasible than ever, to implement at population level*: The historical experience from postwar Europe, Japan, and Korea demonstrated the feasibility of mass radiographic screening, without compromising ‘routine’ TB services [12]. Today, new technologies are increasingly addressing concerns of feasibility. The miniaturization of digital radiography with reduced radiation exposures and availability of portable hand-held X-rays (HHX-rays) equipped with validated artificial intelligence (AI) for automated interpretation is sharply increasing the scalability of mass radiographic screening. The increased awareness and demand for Portable HHX-rays has further reduced costs. These advancements offer the opportunity for higher volumes of X-ray screening than has previously been possible [13], while detection thresholds may also be ‘tuned’ for different transmission settings. Radiographs inevitably identify abnormalities that require follow-up, including TB testing. Near point-of-care assays for detection of TB and drug resistance facilitate on-the-spot diagnosis of persons with abnormal radiographs. India’s National TB Elimination Program, with nearly 9000 Nucleic Acid Amplification Test(NAAT)systems, has saturated Community Health Centres with NAATs in at least half the states in the country.

*Properly implemented, mass screening may have wide population benefits beyond TB*: Mass health screening may include screening for diabetes, hypertension, chronic lung disease, or other diseases of public health importance. Chest radiographs detect a range of abnormalities, which may be attributable to other conditions, including lung malignancies. Overall, the widest public health benefits can only be achieved if health screening and linkage to appropriate care is implemented effectively for diseases of public health importance beyond just TB.

To explore the potential epidemiological impact of mass screening in India, Fig 1 shows projections from a mathematical model of a hypothetical program involving use of an end-to-end locally-made solution set, including portable digital radiographic system [14], automated image interpretation [13], and near-point of care TB assays [15], deployed in each of India’s ~8,500 community development blocks. The model used for these projections, described in further detail in S1 Text, incorporates data from India’s recent prevalence survey [4].

**Fig 1:**
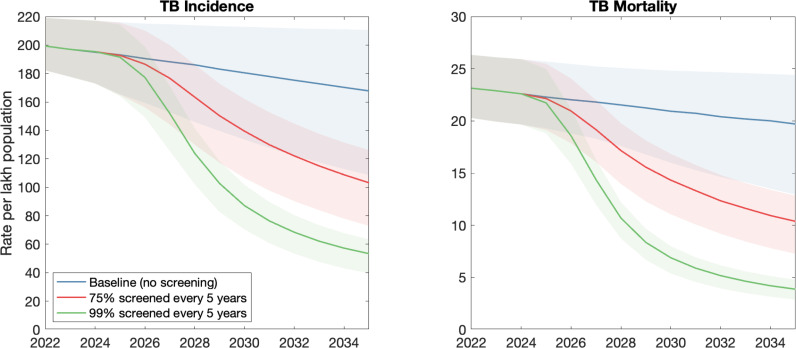
Modelled impact of mass screening for TB in India. We assume an intervention where mobile digital chest X-rays, together with artificial intelligence (AI) reads, are employed to achieve sustained, population-level screening for TB and other diseases. Shown are scenarios where the adult population is screened every 5 years starting from 2025 (green curve), and where 75% of the population is screened over the same period (red curve). In both cases we assumed that the intervention would be scaled up in a linear way, from 2025 to 2028. See supporting technical information for further technical details.

Overall, a ‘maximal’ coverage scenario in which the whole adult population is screened at least once every 5 years would avert 49% of cumulative TB deaths (95% uncertainty interval (UI) 41% – 57%) and 40% of cumulative incidence (95% UI 31% – 47%) between 2025 and 2035. A partial coverage effort (namely, three-quarters of the maximal coverage scenario) would still avert 23% of cumulative TB deaths (95% UI 18% – 29%), and 19% of cumulative incidence (95% UI 13% – 23%) between 2025 and 2035. The ‘maximal screening effort’ shown in Fig 1 (green curve) would require a one-time cost of USD 325 million (2,784 Crore Indian Rupees), and thereafter an average of USD 1.1 billion in running costs per year. The ‘partial coverage effort’ shown in Fig 1 (red curve) would require a one-time cost of USD 240 million (2,054 Crore Indian Rupees), and thereafter an average of USD 830 million in running costs per year. Further information on the costing approach is provided in S1 Text.

The challenge addressed by mass screening – that of persistently high levels of undetected, infectious TB – is by no means unique to India. Prevalence surveys worldwide have highlighted this challenge in other high-burden settings as well. Likewise, although modelled in the context of India, the rationale for mass screening is likely to apply in high-burden settings worldwide. Such an ambitious intervention will of course come with substantial implementation challenges, but existing TB services are highly unlikely to eliminate TB by mid-century. Although those at highest risk of TB might be prioritised in the initial stages of implementation, it will be critical to rapidly extend coverage to the general population, to rapidly quell community transmission from prevalent TB cases. The initial capital expenditure of mass screening seems large, but is far less than alternatives such as a vaccination program with partially-effective vaccines, with health benefits that will take longer to realize.

In any setting, such a mass screening program will require extraordinary political and community commitment, from all levels of society. India has already, however, mobilized the highest levels of political commitment and large swaths of society in the fight against TB; meanwhile, new and emerging tools are making screening more feasible than ever. Appropriately implemented, mass screening offers the most immediate opportunities for addressing TB as a global health problem.

## Supporting information

S1 Text Supporting technical information. (DOCX)
