# Supplementary material for: The potential of community-based radiographic screening to end TB in India
Source: PLOS Glob Public Health. 2025 Jun 24;5(6):e0004716. doi: 10.1371/journal.pgph.0004716 (PMC12186966; doi:10.1371/journal.pgph.0004716)
Supplement: S1 Text — (DOCX) [file pgph.0004716.s001.docx]

**The potential of community-based radiographic screening to end TB in India**

Supporting technical information

**Model specification**

We simulated the deterministic, compartmental model with equations defined as follows.

Uninfected, $U$:

$$\frac{dU}{dt}=b-\lambda U$$

Infected with TB, $L_{F}$:

$$\frac{dL_{F}}{dt}=\lambda U+c\lambda\left( R_{lo}+R_{hi}+R_{stbl} \right)-\left[ u+v+r_{TPT}+\mu\right]L_{F}$$

Infected with TB, $L_{S}$:

$$\frac{dL_{S}}{dt}=v L_{F}-\left[ w+r_{TPT}+\mu\right]L_{S}$$

Infected with TB, $L_{FP}$:

$$\frac{dL_{FP}}{dt}=r_{TPT} L_{F}-\left[ v+u (1-{eff}_{TPT})+\mu\right]L_{FP}$$

Infected with TB, $L_{SP}$:

$$\frac{dL_{SP}}{dt}=r_{TPT} L_{S}+v L_{FP}-\left[ w (1-{eff}_{TPT})+\mu\right]L_{SP}$$

Asymptomatic TB, $I_{A}$:

$$\frac{dI_{A}}{dt}=u L_{F}+w L_{S}+u (1-{eff}_{TPT})L_{FP}+w (1-{eff}_{TPT})L_{SP}-\left( r_{sym}+\sigma+m+\mu\right)I_{A}$$

Symptomatic TB, $I_{S}$:

$$\frac{dI_{S}}{dt}=r_{sym}I_{A}-\left( e+\sigma+m+\mu_{TB} \right)I_{S}$$

Presenting for diagnosis in public $\left( pu \right)$ and private $\left( pr \right)$ sectors, $D^{\left( pu \right)}, D^{\left( pr \right)}:$

$$\frac{dD^{\left( s \right)}}{dt}=p_{cs}^{\left( s \right)}(eA+fE)-\left( \mu_{TB}+\sigma+m+r_{Dx} \right)D^{\left( s \right)}$$

On treatment in public $\left( pu \right)$ and private $\left( pr \right)$ sectors, $T^{\left( pu \right)}, T^{\left( pr \right)}:$

$$\frac{dT^{\left( pu \right)}}{dt}=r_{Dx}p_{Dx}^{\left( pu \right)}D^{\left( pu \right)}+m(I_{A}+I_{S}+D^{\left( s \right)}+E)-\left( \tau+\delta^{\left( pu \right)}+\mu\right)T^{\left( pu \right)}$$

$$\frac{dT^{\left( pr \right)}}{dt}=r_{Dx}p_{Dx}^{\left( pr \right)}D^{\left( pr \right)}-\left( \tau+\delta^{\left( pr \right)}+\mu\right)T^{\left( pr \right)}$$

Temporarily dropped out of careseeking following missed diagnosis, $E$:

$$\frac{dE}{dt}=r_{Dx}\sum_{s} \left( 1-p_{Dx}^{\left( s \right)} \right)D^{\left( s \right)}-\left( f+\sigma+\mu_{TB} \right)E$$

Recovered after treatment non-completion (with high relapse risk), $R_{hi}$:

$$\frac{dR_{hi}}{dt}=\sum_{s} \delta^{\left( s \right)}T^{\left( s \right)}+\sigma(I_{A}+I_{S}+E+\sum_{s} D^{\left( s \right)})-\left( \rho_{hi}+r_{stbl}+c\lambda+\mu\right)R_{hi}$$

Recovered after treatment completion (with low relapse risk), $R_{lo}:$

$$\frac{dR_{lo}}{dt}=\sum_{s} \tau T^{\left( s \right)}-\left( \rho_{lo}+r_{stbl}+c\lambda+\mu\right)R_{hi}$$

Recovered with long-term relapse risk, $R$:

$$\frac{dR}{dt}=r_{stbl}\left( R_{hi}+R_{lo} \right)-(\rho_{stbl}+c\lambda+\mu)R$$

Force-of-infection, $\lambda:$

$$\lambda=\beta\left[ \rho I_{A}+I_{S}+E+\sum_{s} D^{\left( s \right)} \right]$$

The model was calibrated to a range of epidemiological and programmatic data, including: WHO estimates for incidence and mortality rates in India; data from India’s recent national tuberculosis prevalence survey on the proportion of prevalent TB that was asymptomatic, and for the proportion symptomatic who had not sought care; and notification data. Calibration was performed using Bayesian Markov Chain Monte Carlo (MCMC), to propagate uncertainty from model inputs to model projections. After removing the burn-in and ‘thinning’, 250 samples from the posterior density were used to create model simulations. In all model projections, central estimates were produced using 50^th^ percentiles, while 2.5^th^ and 97.5^th^ percentiles were used for uncertainty intervals.

**Costing analysis**

*Overview of the costing approach*

We used an ingredients-based approach to estimate intervention costs, with unit costs derived through field visits and virtual calls with program person, field visit in rural and urban areas, interviews with field staff, NGO conducting X-ray camps, and staff involved in current X-ray screening intervention in India (the latter including supervisor, radiographer and paramedical worker). Unit cost were estimated based on the available documents, market-rates and norms used in India’s National Tuberculosis Elimination Programme (NTEP). Estimated output and coverage per screening team, per field team (door-to-door) visits and per camp-output is based on the discussions with the involved staff and previous program experience.

We assumed the following: a screening unit, consisting of an ultraportable X-ray machine unit with its accessories, would be placed at the District level (at least 1 per District). Every unit would have two full time staff: a radiographer or paramedical worker trained to operate and maintain the machine, and supportive staff for carrying the machine and ensuring the patient and X-ray plate are in the proper position during X-ray exposure. The screening unit will have a hired vehicle (4-wheeler) for mobility. Ultraportable X-ray machines will have AI with both offline and online facility to read the X-ray and identify abnormal X-rays for further investigations.

We further assumed that NTEP will systematically plan to cover block-wise / ward-wise / village-wise geography to cover the eligible population ($\geq$15 years old). The population would be mobilized with door-to-door field visit by a 2-membered (local volunteer / ASHA healthcare worker) who would invite eligible individuals ($\geq$15 years) to come to the X-ray camp in the community, or nearby health facility, in the same area. Camps would be organized with support from local health system field staff or health facility staff, and were assumed to be utilized for chest X-ray for an average of 70 persons per day in rural areas and 100 persons per day in urban area. We assumed that 250 camps (125 in community and 125 in health facilities) would be organized annually. Community camps would be organized with the support of local health system staff at a community place such as community hall or ‘gram panchayat’. The activity would be embedded within the TB program and would be covered for planning, training and routine supervision with the existing program staff.

Other assumptions included the following:

- We considered the district-wise population as of 2023, drawing from NTEP demographic data.
- The proportion of population being over 15 years age was considered as 75% (World bank data) with household size 4.1 (GoI – MOSP)
- 36% of India’s population are urban (World bank data)
- Two membered team (local volunteers/ASHA healthcare workers) already working for the health system will conduct door-to-door visits to cover 40 houses in rural areas and 60 houses in urban areas, considering the population density, and will provide a slip to attend X-ray/screening camp to be organized in the nearby community, or facility closest to the home.
- On average, 70 people would be screened with chest X-ray per camp in rural areas, and 100 per camp in urban areas.
- Each screening unit would hold 250 camps per year, half in the community and half in facilities.
- Each screening unit would consist of one ultra-portable X-ray machine with AI (either online offline) to read unlimited scans with one laptop, one tablet device with internet connectivity with two full-time people, along with one-four wheeler (hired) for mobility.
- Mass radiology screening of population intervention would be embedded within the program and program structures. Existing man-power would be utilized for planning, training, supervision & monitoring and recording-reporting.
